# Two‐Stage Recruitment Design to Reduce Magnetic Resonance Imaging Screening Cost for a Theoretical Clinical Trial of White Matter Hyperintensity Progression

**DOI:** 10.1161/JAHA.124.036140

**Published:** 2024-11-15

**Authors:** Marco Egle, Mohini Johri, Melinda C. Power, Jennifer A. Deal, Clifford R. Jack, Kevin J. Sullivan, Thomas H. Mosley, Rebecca F. Gottesman

**Affiliations:** ^1^ National Institute of Neurological Disorders and Stroke, Intramural Research Program National Institutes of Health Bethesda MD USA; ^2^ Department of Epidemiology George Washington University Milken Institute School of Public Health Washington DC USA; ^3^ Department of Epidemiology Johns Hopkins Bloomberg School of Public Health Baltimore MD USA; ^4^ Department of Radiology Mayo Clinic Rochester MN USA; ^5^ Department of Medicine University of Mississippi Medical Center Jackson MS USA

**Keywords:** clinical trial, recruitment, retinal measures, sample size, white matter hyperintensity, Magnetic Resonance Imaging (MRI), Cardiovascular Disease, Cerebrovascular Disease/Stroke

## Abstract

**Background:**

White matter hyperintensities (WMH) and their progression are associated with risk of dementia and stroke, so are an important target for clinical trials. The cost of broad magnetic resonance imaging (MRI) screening to identify eligible individuals, however, limits the feasibility of designing clinical trials targeting WMH. A low‐cost retinal or clinical screening measure before MRI could reduce recruitment costs versus an MRI‐only screening design in a hypothetical clinical trial.

**Methods and Results:**

Data from the Atherosclerosis Risk in Communities study with valid retinal and WMH measurements (*N*=1311) were used. To identify a population at greater likelihood of significant WMH on MRI and thus reduce the number of screening MRIs required, we evaluated 3 theoretical prescreening measures: (1) retinal, (2) clinical, (3) combined clinical‐retinal. Given a target sample for clinical trials (*N*=646), we calculated screening sample sizes based on the proportion within the population having an elevated score for each prescreening measure (separately) multiplied by the proportion of significant WMH among those with that prescreening feature. Recruitment costs were calculated using estimated retinal and MRI cost estimates. Compared with the estimated cost of MRI‐only screening (>$4.24 million, requiring MRI on 6526 participants), prescreening for a high clinical score resulted in total cost of $2.47 million, with an initial screening group of 52 778 participants, with MRI in 3801. A high clinical‐retinal score cutoff resulted in costs of $2.9 million while requiring 13 572 participants, with 3801 completing MRI.

**Conclusions:**

A 2‐stage design with low‐cost prescreening measures is a promising approach, resulting in reduced theoretical recruitment costs compared with an MRI‐only design.

**Nonstandard Abbreviations and Acronyms**
ARIC: Atherosclerosis Risk in Communities
CHS: Cardiovascular Health Study
FP: false positive
TP: true positive
WMH: white matter hyperintensities

**Research Perspective**

**What Is New?**

- Can a retinal risk score, a clinical risk score, or a combination of the 2 be used as a prescreening measure for a theoretical 2‐stage retinal‐magnetic resonance imaging recruitment design to inform potential future trials aimed at recruiting individuals at high risk for white matter hyperintensity progression?

**What Question Should Be Addressed Next?**

- Can innovative machine‐learning approaches on low‐cost prescreening measures further reduce recruitment costs for clinical trials in cerebrovascular disease?

White matter hyperintensity (WMH), a common radiological feature in age‐related sporadic cerebral small vessel disease, can be detected as bilateral hyper‐intense regions on a T2 or fluid‐attenuated inversion recovery MRI scan.^1^ WMH is usually associated with aging: estimates of the prevalence of WMH in individuals above age 65 years have varied depending on the methodology and the clinical characteristics of the selected sample, ranging between 20% and 87%.^1^, ^2^, ^3^ In some individuals, however, significant WMH lesions are already apparent in late midlife (estimated frequency between 6% and 51%),^4^, ^5^, ^6^, ^7^ often in the setting of hypertension and genetic predisposition. Their presence in late midlife has been associated with impaired cognitive function and with cognitive decline, as well as with an increased lifetime risk of stroke and mortality.^8^, ^9^, ^10^, ^11^ Thus, clinical trials aimed at slowing down WMH lesion growth during this time window are of great interest, given the sequelae of these lesions and their progression.^12^

One significant challenge in designing clinical trials in individuals with WMH and with a high risk of lesion progression is to recruit the right population in late midlife without having to spend large financial resources during screening. As brain imaging is an expensive modality to screen for WMH lesion burden, more cost‐effective prescreening approaches could be used to identify those at highest risk of having this imaging finding. Retinal microvascular imaging, for instance, may be a promising prescreening tool available at a relatively low cost. Previous evidence in the ARIC (Atherosclerosis Risk in Communities) study has shown that, cross‐sectionally, individuals with retinopathy in late midlife were more than twice as likely to have significant WMH lesions than those without retinopathy.^13^ Furthermore, arteriovenous nicking and retinopathy were significantly associated with more volumetric WMH progression over 10 years.^14^ These studies emphasize the potential value of retinal imaging in identifying people with WMH and those at risk of WMH progression. Clinically available patient information also may have value—and is even more cost effective—in the prescreening process to identify those at greatest risk of significant WMH. Previous evidence has shown that cardiovascular risk scores in midlife were associated with greater WMH volume and progression.^15^

In this study, our aim was to determine whether a retinal risk score, which was our primary prescreening measure, a clinical risk score, or a combination of the 2 can be used as a prescreening measure for a theoretical 2‐stage retinal‐magnetic resonance imaging (MRI) recruitment design to inform potential future trials aimed at recruiting individuals at high risk for WMH progression (Figure 1). Recruited participants in late midlife would be screened on the presence of retinal signs, clinical features, or their combination in a first stage, with confirmatory MRI in the highest risk subgroup for inclusion/exclusion decisions in a second stage; we hypothesized that this model would be theoretically cheaper than direct screening with MRI in a broader population. Given a specified minimum sample size for a phase‐2 clinical trial, we evaluated, using real‐life epidemiologic data from the ARIC study, how different screening approaches affect a theoretical study's recruitment costs and its ability to detect significant WMH progression over 10 years. We furthermore explored whether a weighted screening approach more sensitive to WMH burden would more effectively reduce recruitment costs than the screening measures initially selected for the primary analysis. Finally, we determined whether a 2‐stage design would reduce the overall recruitment costs not only in a late midlife but also in a late‐life population.

**Figure 1:**
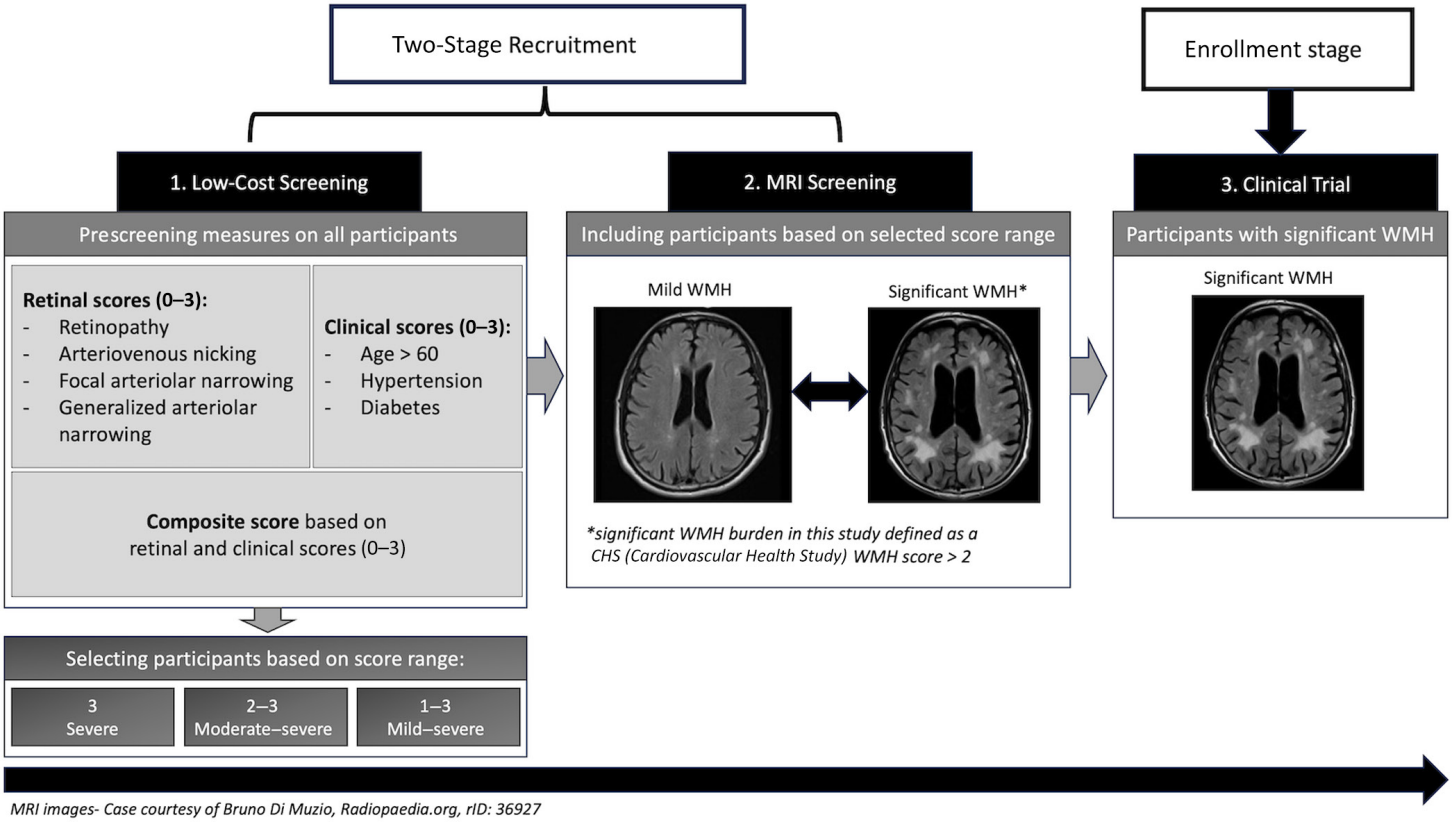
Overview of the 2‐stage recruitment design. MRI indicates magnetic resonance imaging; and WMH, white matter hyperintensities.

## METHODS

The full data are not available publicly because of participant privacy and consent. More information regarding the data is available from the corresponding author on reasonable request.

The study was approved by each institutional review board. The study was performed in accordance with the ethical standards as laid down in the 1964 Declaration of Helsinki and its later amendments or comparable ethical standards.

### Study Population

We used real‐life epidemiologic data from the prospective ARIC cohort to inform our calculations. The ARIC study is an ongoing community‐based prospective study of 15 792 participants aged 45 to 64 years at baseline (1987–1989) from 4 communities across the United States. Between baseline and the year 2013, there were multiple in‐person visits in the years 1990 to 1992 (visit 2), 1993 to 1995 (visit 3), 1996 to 1998 (visit 4) in midlife and 1 follow‐up visit in the years 2011 to 2013 in late life (visit 5).^16^

### Visit 3 MRI (Year 1993–95)

Of the 12 887 participants seen at visit 3 in their late midlife (age range 51–70 years), a subset of 1930 individuals from 2 ARIC sites who were >55 years old and who had no contraindications for MRI (ie, metal or electrical implanted devices, occupations with exposure to metal fragments, aneurysm brain surgery) underwent MRI as part of the ARIC Brain MRI ancillary study.^17^ Of those screened, 2% of women and 6% of men were not eligible. Proton‐density weighted images were graded for WMH severity on the CHS (Cardiovascular Health Study) scale at the ARIC MRI Reading Center at Johns Hopkins Medical Insitutions. The grading was performed blinded to the individuals' clinical characteristics and 1925 participants had graded WMH measures. As previously reported, interrater reliability for graded WMH in ARIC was good, κ=0.76.^17^ In this study, visit 3 was considered as the baseline MRI assessment (Figure S1). Quantitative volumetric brain imaging was not available for the baseline MRI scans. A CHS rating >2 was considered consistent with significant WMH.

### Follow‐Up MRI (Year 2004–06)

Between 2004 and 2006, as part of the ARIC Brain MRI ancillary study, 1134 of those individuals previously scanned at visit 3 underwent a follow‐up MRI scan^17^; for the purposes of this article, to allow consistency relative to other ARIC articles, this visit (and its associated MRI scan) will be referred to as visit 4B (it took place between ARIC visit 4 and ARIC visit 5) (Figure S1). MRI images were graded, again using the CHS rating scale, at the University of Washington by several neuroradiologists (blinded to clinical information and to the baseline MRI scan) who were trained by one of the readers involved in the baseline study.^17^

In addition to the visual WMH severity grading as at visit 3, a semiautomated volumetric WMH measure was also calculated based on the fluid‐attenuated inversion recovery images, which was standardized to a total intracranial volume of 1500 cm^3^.^18^ Because everyone with a visit 4B MRI had both a CHS scale measure and a volumetric measurement of WMH, the relationship between these 2 measures was used to estimate a quadratic regression equation allowing conversion between CHS scale and volumetric measurement of WMH. This regression was used to convert visit 3 CHS ratings to estimated volumes to allow for analysis of progression.

### Visit 5 MRI (Year 2011–13)

Of the 6538 participants in late life (age range of 71–90 years) seen in person at ARIC visit 5 (the ARIC Neurocognitive Study), 1978 individuals underwent MRI assessment, across all 4 ARIC sites. Participants were selected for MRI on the basis of (1) having had a prior research MRI as part of ARIC visit 3, (2) having evidence of cognitive decline, or (3) a random age‐stratified sample of the remaining individuals, and (4) not having contraindications to MRI.^19^ Images were reviewed and analyzed centrally at the Mayo Clinic MRI Reading Center blinded to clinical information. Of these, 1971 individuals at visit 5 had scans substantively of sufficient quality for WMH volume measurement, which was quantified on T2 fluid‐attenuated inversion recovery sequences and measured using an algorithm developed at the Mayo Clinic in Rochester, Minnesota.^20^ We determined that significant WMH burden (as defined by a CHS >2, which we used for the visit 3 threshold for significant WMH) would be equivalent to a WMH volume ≥9.3 cm^3^ based on previous study estimates.^21^ The same theoretical recruitment analysis as previously performed for visit 3 (late midlife) was then carried out for visit 5 (late life) using this threshold. An overview about the ARIC study design and the respective MRI assessments can be found in Figure S1.

### Retinal Variables

Fundus photography was conducted on all participants seen at visit 3 and at visit 5 as described elsewhere.^22^ The presence of retinal lesions was assessed using the modified Airlie House classification, as used in the ETDRS (Early Treatment Diabetic Retinopathy Study). Retinopathy was classified as present (level =14–87) versus absent (level =10–13). Arteriovenous nicking was considered as “definite” when at least 1 venous blood column was tapered on both sides of its crossing underneath an arteriole. Focal arteriolar narrowing was determined as “definite” based on the grading and number of arterioles estimated to be ≥50 μm in diameter with a constricted area less than two thirds the width of proximal and distal vessel segments. Generalized arteriolar narrowing was defined as the lowest quartile of the central retinal arteriolar equivalent, which was quantified on the arteriolar diameters within a prespecified zone surrounding the optic nerve. All photographs were assessed by certified graders at a central reading center who were masked to participants' characteristics.

### Clinical and Demographic Variables

Demographic information such as date of birth, to calculate age, was collected at the baseline visit (1987–1989). Several clinical features were measured at visit 3 (1993–1995) and visit 5 (2011–2013) including hypertension, defined as systolic blood pressure >140 mm Hg, diastolic blood pressure >90 mm Hg or use of antihypertensive medications, and diabetes, defined as fasting glucose level ≥126 mg/dL, nonfasting glucose level ≥200 mg/dL, self‐report of physician‐diagnosed diabetes, or use of oral diabetes medications or insulin.

### Statistical Analysis

#### Computation of the Prescreening Measures

Prescreening measures were separately calculated for late midlife (visit 3) and late life (visit 5).

Our primary prescreening measure was a novel comprehensive retinal score, ranging from 0 to 3: this was generated based on the presence versus absence of each of 4 retinal signs.^24^ The individual retinal characteristics were scored as follows: Presence of retinopathy=3; presence of arteriovenous nicking=1; presence of focal arteriolar narrowing=1; presence of generalized arteriolar narrowing=1. Participants with a summed score of >3 were given the highest possible retinal score of 3 because retinopathy is seen to represent end‐stage retinal disease (so it would not be possible to have more than 3 points, as these other findings usually would precede retinopathy).^25^ Similarly, a clinical scoring system, which was our secondary prescreening measure and served as comparison, was created, which ranged from 0 to 3. The presence of each of the 3 demographic and clinical risk factors, that is age (>60 years old), hypertension, and diabetes, was given 1 point, and summed up to form a composite score per participant. To form a similar meaningful clinical score for late life at visit 5, the age factor was modified and a cutoff score of age 78 years was used (18 years were added to the cutoff score from visit 3 as visit 5 took place 18 years later). In addition, a combined clinical‐retinal scoring system, ranging from 0 to 3, combined the clinical as well as the retinal features. The maximum clinical‐retinal score remained at 3 for consistency and ease of comparison but was otherwise the sum of the clinical and retinal scores unless >3, in which case it was truncated at 3. The scores were graded based on severity as follows: none (score=0), mild (score=1); moderate (score=2); severe (score=3). Given that the various retinal and clinical prescreening features may have different predictive values for the presence of WMH, a weighted version of the 3 prescreening scoring systems was also created in the secondary analysis (Figure S2). Details regarding the computation of the weighted scores are given in Data S1.

#### Single and 2‐Stage Recruitment Computation for Late Midlife and Late Life

To estimate the hypothetical recruitment sample size and cost at visit 3 (late midlife) and visit 5 (late life), several measures had to be specified before the computation. First, a minimum sample size estimate for a phase‐2 clinical trial had to be formulated. Based on prior evidence, we chose a minimum target sample size estimate of 646 participants with sporadic cerebral small vessel disease for a theoretical clinical trial.^26^ Second, we estimated the standard costs of the retinal fundus and magnetic resonance imaging for each participant to be $32.50 and $650 respectively.

**Figure 2:**
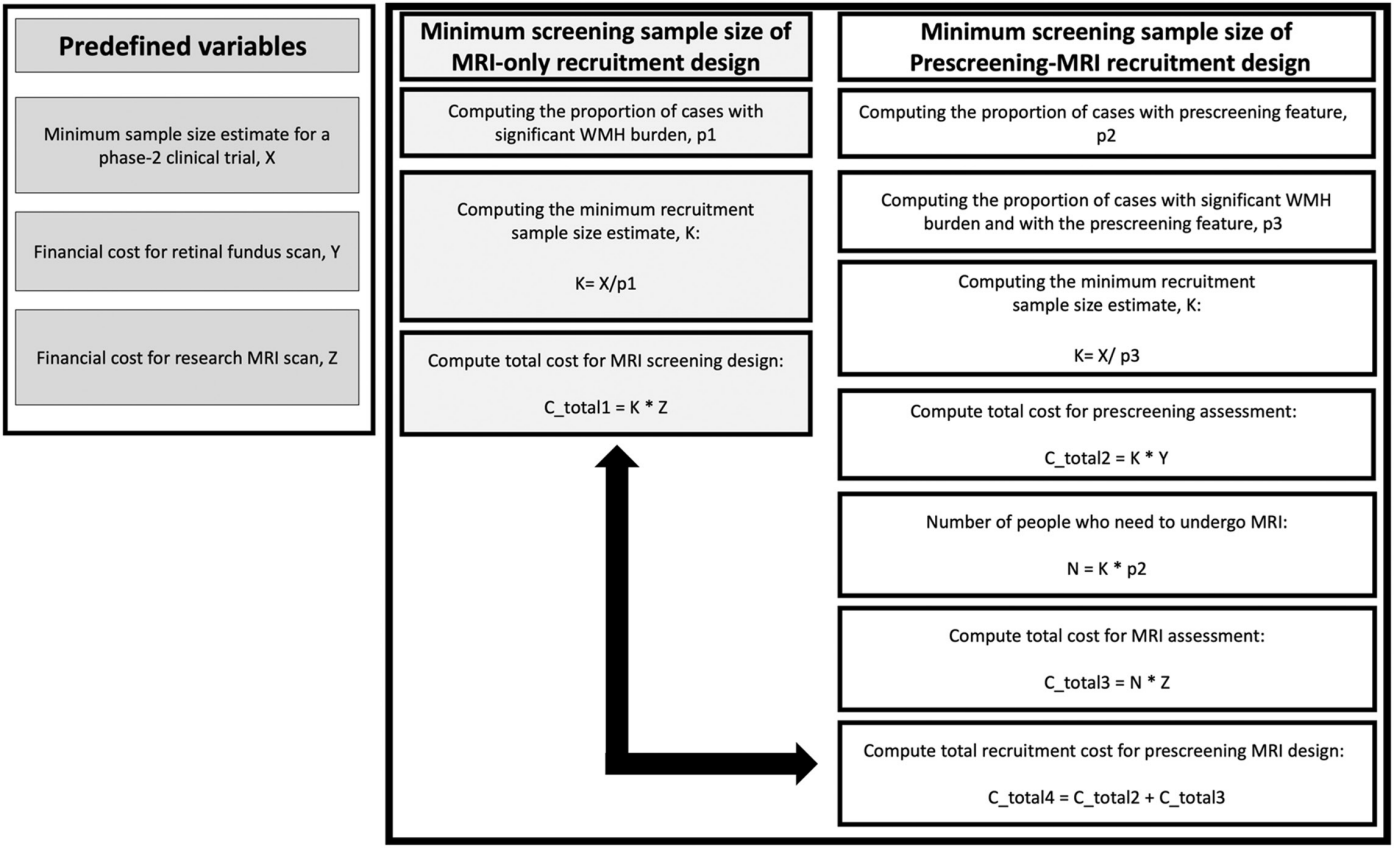
Flow diagram demonstrating the computation of recruitment size and cost in the MRI‐only and the prescreening‐MRI recruitment design. MRI indicates magnetic resonance imaging; and WMH, white matter hyperintensities.

The computations of the various recruitment designs are illustrated in Figure 2. In the MRI‐only recruitment design, the minimum sample size estimate was divided by the proportion of participants with significant WMH burden (CHS >2) to estimate the number of participants required at the recruitment stage. The total MRI screening cost was calculated by multiplying the number of participants at the recruitment stage with the research cost for a single MRI scan.

In the prescreening‐MRI recruitment design, different score ranges were used to select participants at the prescreening stage. They were labeled as follows to reflect the retinal and clinical burden: severe: score of 3; moderate–severe: score of 2 to 3; mild–severe: score of 1 to 3. The proportion of individuals with scores in each given range was estimated, and then, within each of those groups, the proportion having significant WMH was calculated. When these were multiplied, this yielded a proportion with significant WMH, the inverse of which was multiplied by the overall goal clinical trial sample size to yield that total recruitment sample size for each score category. The total recruitment sample size was then multiplied by the financial cost of a single retinal prescreening assessment, $32.50, to estimate the overall prescreening costs. For the clinical prescreening measure, no cost was assigned as determination of hypertension and diabetes status was felt to be part of standard clinical care. To determine the number of participants who would need to undergo MRI at a second screening stage, the proportion of participants with a chosen prescreening score was multiplied by the total recruitment sample size. The MRI cost of the 2‐stage design was estimated by multiplying the number of participants required to undergo MRI with the cost for a single research MRI scan, $650. The total cost of the prescreening‐MRI recruitment design was then computed by adding the prescreening and the MRI screening costs together. To assess how well the different prescreening scores of varying severity predict WMH burden, we also computed several performance metrics including the true positive (TP; sensitivity) and false positive (FP) rate for the unweighted as well as weighted scores. We computed the FP rate as this metric would be important to explain the greater number of MRI scans required and the higher MRI screening costs for certain prescreening modalities and severity ranges.

#### Longitudinal Analysis on WMH Progression

WMH volume was not directly measured volumetrically and was therefore not available at visit 3. To estimate the 10‐year WMH volume change in those participants with repeat MRI assessment, starting in late midlife, the CHS scores at baseline were transformed into WMH volume using a previously published prediction quadratic equation (*R* ^2^=0.80) (based on visit 4B [2004–2006] data where all MRI scans were analyzed with both methods), and the difference in WMH volume at baseline and at the follow‐up time point (when volumetric measurements were available) was computed.^18^ Employing a Kruskal–Wallis test, differences in volume change between the various prescreening scores (0–3) were tested. Planned post hoc comparisons tested for differences in the mild, moderate, or severe scores in the prescreening stage.

#### Attrition Rate on 2‐Stage Recruitment Estimation

To determine how attrition would affect the total recruitment sample size of the 2‐stage design, different rates (10%, 20%, 30%) were also considered in the 2‐stage computation. To calculate the recruitment sample size adjusted by attrition, the proportion of participants with the prescreening feature and with significant WMH burden was multiplied by the proportion of people who would eventually be enrolled in the clinical trial (attrition proportion). The target sample size (*N*=646) was then divided by the product to estimate the overall recruitment sample size.

#### The 2‐Stage Recruitment Calculator

An open Shiny webapp for calculating the 2‐stage recruitment sample size and cost for any population is available at https://marcoegle123.shinyapps.io/2_stage_recruitment_webapp/. Different cost estimates, target sample sizes, and attrition rates can be specified in the program. We think that this program may particularly be useful when aiming to reduce screening costs in a population where only a small proportion of individuals has an imaging characteristic, which would make them eligible for clinical trial enrollment, and where at the same time a less expensive marker is available which could be used for prescreening. Applications for this design include cerebrovascular diseases and cardiovascular diseases but also cancer and metabolic diseases, especially when designing a clinical trial in a midlife population where only a fraction of the population would be eligible for enrollment. In addition to all the calculating features described in this article, the Shiny web app can also compute the prescreening sample size needed when planning to enroll 2 separate groups of individuals (*optional feature*), for example, male versus female, with a different feature prevalence at the prescreening stage for who should be equally represented at the MRI screening stage. There may be biological and social reasons why the prevalence is lower in one group versus the other.^27^ This feature will help to address differences in willingness to be recruited or differences in disease prevalence. Employing this option, the estimated total recruitment size is computed separately for each of the 2 groups and weights are assigned to recruit more individuals from the group that has a lower feature prescreening prevalence. The user interface of the 2‐stage recruitment app is shown in Data S2.

## RESULTS

### Cohort Characteristics in Late Midlife

Of the 1925 participants with a visit 3 measure of WMH burden, 614 were excluded because of missing and ungradable retinal measures (Figure S3). Excluded participants were older (median age 64 versus 61 years) and more likely to be of Black race (59% versus 45%) and to have diabetes (22% versus 17%) as well as hypertension (53% versus 47%) (Table S1). In 12 participants, clinical information of hypertension or diabetes was imputed via multivariate imputation by chained equations. The single imputed data set consisted of a final study population of 1311 of whom 130 (9.9%) had significant WMH burden, defined as CHS >2 (Table 1). Follow‐up MRI with a valid measure of WMH burden was available in 732 individuals (Figure S3). Individuals with significant WMH burden (versus without) had significantly higher rates of prevalent retinopathy (17% versus 6.2%), arteriovenous nicking (15% versus 5%), focal arteriolar narrowing (19% versus 11%), hypertension (66% versus 45%), and being older than 60 years of age (82% versus 63%). Participants with significant WMH burden (CHS score >2) compared with those with mild or no WMH burden also had a significantly greater prevalence of severe retinal, clinical, and clinical‐retinal score categories (Table 1).

**Table 1:** Features of the Individual Measures and Summary Scores in Late Midlife, by WMH Category

Characteristics	Overall, N=1311	No or low WMH burden, N=1181	Significant WMH burden, N=130	*P* value‡
Age, y*	61 (58,65)	61 (58,65)	64 (61,68)	<0.001||
Retinal measures†				
Retinopathy	95 (7.2%)	73 (6.2%)	22 (17%)	<0.001||
Arteriovenous nicking	78 (5.9%)	59 (5.0%)	19 (15%)	<0.001||
Focal arteriolar narrowing	157 (12%)	132 (11%)	25 (19%)	0.007||
Generalized arteriolar narrowing	324 (25%)	291 (25%)	33 (25%)	0.9
Demographic and clinical measures†				
Age ≥60 y	851 (65%)	745 (63%)	106 (82%)	<0.001||
Diabetes	219 (17%)	192 (16%)	27 (21%)	0.2
Hypertension	618 (47%)	532 (45%)	86 (66%)	<0.001||
Unweighted summary scores† ^,^ §				
Retinal score				<0.001||
None	781 (60%)	721 (61%)	60 (46%)	
Mild	363 (28%)	325 (28%)	38 (29%)	
Moderate	69 (5.3%)	60 (5.1%)	9 (6.9%)	
Severe	98 (7.5%)	75 (6.4%)	23 (18%)	
Clinical score				<0.001||
None	237 (18%)	231 (20%)	6 (4.6%)	
Mild	554 (42%)	509 (43%)	45 (35%)	
Moderate	426 (32%)	363 (31%)	63 (48%)	
Severe	94 (7.2%)	78 (6.6%)	16 (12%)	
Clinical‐retinal score				<0.001||
None	171 (13%)	169 (14%)	2 (1.5%)	
Mild	396 (30%)	367 (31%)	29 (22%)	
Moderate	378 (29%)	341 (29%)	37 (28%)	
Severe	366 (28%)	304 (26%)	62 (48%)	

WMH indicates white matter hyperintensities.

* Median (Q1, Q3).

^†^ n (%).

^‡^ Wilcoxon rank sum test, Pearson's Chi‐squared test comparing significant vs no or low WMH burden.

^§^ Score categories: none = 0; mild = 1, moderate = 2, severe = 3 or highest score.

^||^ p‐value significance level < 0.05.

### Primary Analysis: Recruitment Estimation in Late Midlife with Unweighted Scores

Only a small proportion of individuals in late midlife had significant WMH burden (9.9%). When the goal sample size (*N*=646) was divided by this proportion, the results showed that a total of 6526 participants would be needed at the recruitment stage for the MRI‐only recruitment design. Assuming an MRI cost of $650 per individual, the total associated recruitment cost would be $4.24 million (Table 2).

**Table 2:** Estimated Sample Size for a Theoretical Recruitment Study by Each of the Unweighted Screening Approaches in Midlife

Theoretical inclusion criteria	Overall		1. Prescreening stage		2. MRI screening stage	
			Number of participants at prescreening stage and associated cost (proportion of those individuals with a positive prescreening feature)		Number of participants at the MRI screening stage and associated cost (proportion of those individuals with significant WMH burden)	
Retinal score range	Sample size	Financial cost	Sample size	Financial cost	Sample size	Financial cost
Severe	37 450	$3 042 975	37 450 (7.5%)	$1 217 125	2809 (23%)	$1 825 850
Moderate–severe	26 154	$3 060 655	26 154 (13%)	$850 005	3401 (19%)	$2 210 650
Mild–severe	12 424	$3 634 280	12 424 (40%)	$403 780	4970 (13%)	$3 230 500
Clinical score range						
Severe	52 778	$2 470 650	52 778 (7.2%)	$0	3801 (17%)	$2 470 650
Moderate–severe	10 767	$2 799 550	10 767 (40%)	$0	4307 (15%)	$2 799 550
Mild–severe	6566	$3 500 250	6566 (82%)	$0	5385 (12%)	$3 500 250
Clinical‐retinal score range						
Severe	13 572	$2 911 740	13 572 (28%)	$441 090	3801 (17%)	$2 470 650
Moderate–severe	8718	$3 513 835	8718 (57%)	$283 335	4970 (13%)	$3 230 500
Mild–severe	6751	$4 037 508	6750 (87%)	$219 375	5873 (11%)	$3 817 450
No prescreening						
MRI only	6526	$4 241 900	…	…	6526 (9.9%)	$4 241 900

MRI indicates magnetic resonance imaging; and WMH, white matter hyperintensities.

**Figure 3:**
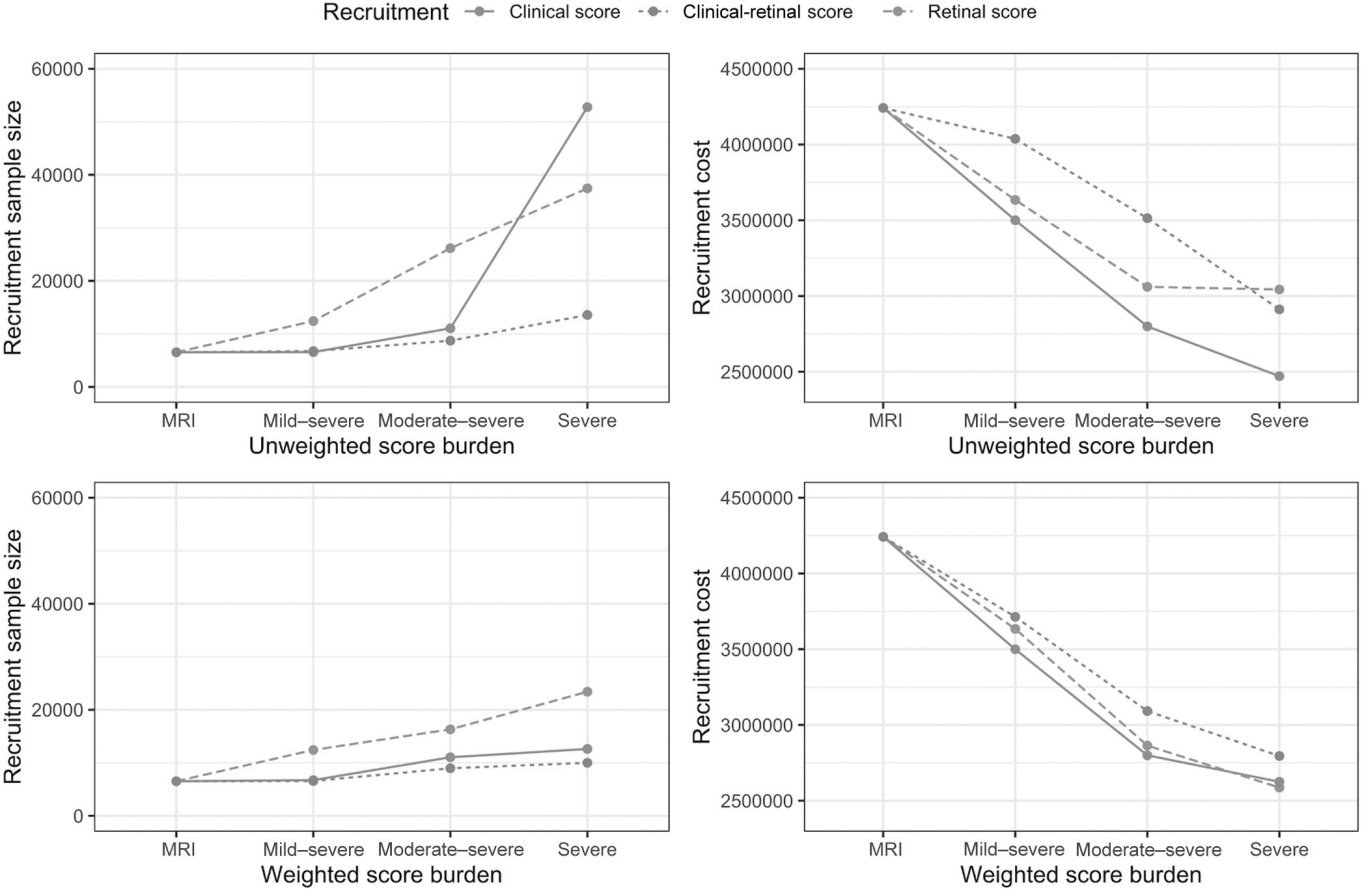
Total recruitment sample size and cost for various score cutoffs for each of the recruitment modalities in midlife. MRI indicates magnetic resonance imaging; and WMH, white matter hyperintensities.

The recruitment cost estimates were significantly lower in all variants of the 2‐stage design (Table 2; Figure 3). For instance, if using a retinal prescreen and selecting only participants with a severe retinal score, 7.5% of individuals would be selected for an MRI scan, and 23% of those individuals entering the MRI stage are estimated to show significant WMH lesions. Thus, to achieve the clinical trial's target sample size (*N*=646), a total of 37 450 participants would therefore be needed to identify 2809 people (7.5% of 37 450) eligible for the MRI stage, of whom 646 (23% of 2809) would be eligible for theoretical trial enrollment. Assuming an MRI cost of $650 and retinal scan cost of $32.50, the total recruitment cost would be $3.04 million of which the MRI cost would be $1.83M.

When comparing the different prescreening modalities, the total recruitment cost was lowest ($2.47 million) when using the clinical score prescreen, and choosing participants of the highest clinical score category who had hypertension and diabetes and who were older than 60 years of age (Table 2; Figure 2). When using this recruitment design, however, 52 778 participants would need to be included at the prescreening stage. Finally, using the combined clinical‐retinal screen, a high clinical‐retinal score cutoff marginally increased costs to $2.9 million while requiring only 13 572 screened participants (Table 2). When examining the TP and FP rate for each of the prescreening modalities, we saw that the TP rate was very high for mild–severe clinical (TP rate=0.95) and for clinical‐retinal (TP rate=0.99) severity ranges (Table S2; Figure S4). However, tFP rate was also high for mild–severe clinical (FP rate=0.80) and for mild–severe clinical‐retinal prescreening (FP rate=0.86). The FP rate was lower for the retinal measures (FP rate=0.06–0.39) or when selecting a severe prescreening range (FP rate=0.06–0.26), which contributed to lower recruitment costs, specifically at the MRI screening stage in late midlife.

When adding attrition rate (10%, 20%, or 30%) to the 2‐stage design, the overall sample size and cost significantly increased as would be expected (Table S3).

### Longitudinal Analysis on WMH Progression

**Figure 4:**
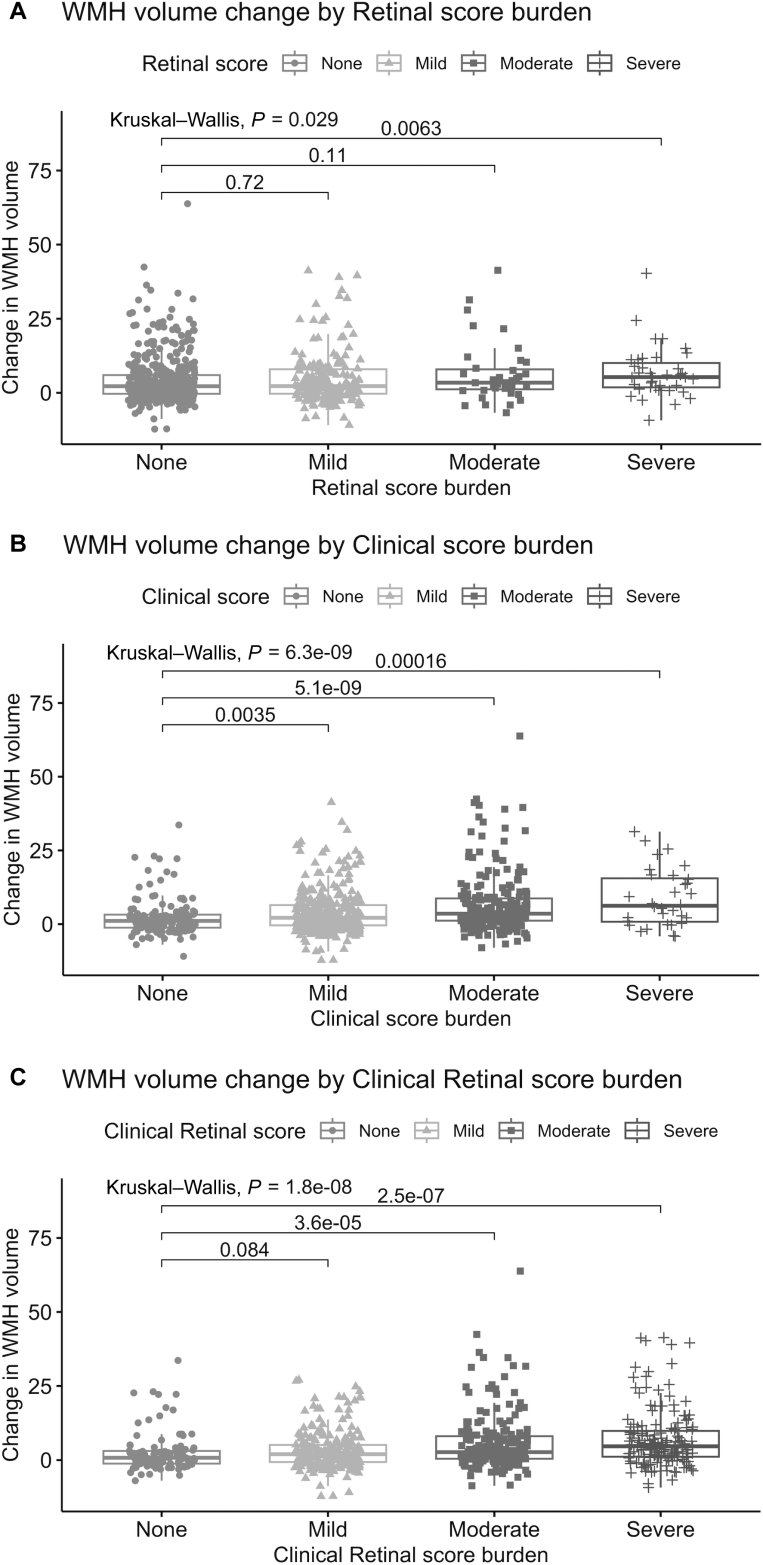
Comparing the 10‐y change in WMH volume (cm^3^) by severity in each of the unweighted prescreening modalities: (A) retinal score burden, (B) clinical score burden, and (C) clinical‐retinal score burden. WMH indicates white matter hyperintensities.

The longitudinal results showed that in all 3 unweighted prescreening designs, participants in the severe score category had more WMH progression than people without any retinal or clinical features (Figure 4; Table S4). In the analyses using a clinical or clinical‐retinal prescreen, significant differences in WMH progression were also found for the moderate prescreening score categories.

### Secondary Analysis: Recruitment Estimation in Late Midlife With Weighted Scores

To evaluate the relative contributions of each component of the prescreen score, we evaluated regularized regression models; in the model with retinal measures as predictors only, retinopathy (*w*=1.210) and arteriovenous nicking (*w*=1.187) had the highest normalized weights. In the clinical model, age 60 years or older (*w*=1.354) and hypertension (*w*=1.314) had the highest weights because they were the strongest predictors in the model (Table S5). As described previously, scores out of a maximum of 3 were generated based on these weights for each of the prescreening approaches. Similar to the results with the unweighted scores, participants with significant WMH burden (CHS >2) had more severe retinal, clinical, and clinical‐retinal weighted scores than those with no or few WMH (CHS≤2) (Table S6).

Using the weighted prescreening measures in the 2‐stage design also significantly reduced the financial costs compared with an MRI‐only recruitment design (Table S7). For instance, to achieve a target sample size of 646 participants and when selecting the highest retinal score as a prescreening measure, 23 406 participants would need to undergo retinal screening, of whom 2809 would undergo MRI screening. The total estimated cost of this 2‐stage design would be $2.59 million and would therefore be $1.65 million lower than the MRI‐only recruitment design ($4.24 million). Interestingly, a lower total recruitment sample size was needed when using the weighted instead of the unweighted prescreening approach (Table 2; Figure 3; Table S7). Whereas 37 450 participants were needed in the severe unweighted retinal category, a total of only 23 406 individuals were required for the same weighted category. The weighted and unweighted recruitment estimates included in the same graph can be found in Figure S5. Similar to the unweighted scores, we observed that the TP rate was particularly high for the mild–severe clinical (TP rate=0.94) and clinical‐retinal (TP rate=0.96) pre‐screening range. At the same time, the FP rate was also high for the weighted mild–severe clinical (FP rate=0.78) and clinical‐retinal (FP rate=0.80) prescreening range. As for the unweighted scores, the TP and FP rate was significantly lower for the retinal prescreening measures (FP rate=0.11–0.39) or when selecting a severe prescreening score range (FP rate=0.11–0.35) (Table S2; Figure S4).

### Cohort Characteristics in Late Life

Based on previous estimates, we defined greater WMH burden (CHS >2) as a continuous WMH volume ≥9.3 cmp For the analysis of a late‐life retinal and clinical prescreen, characteristics of participants with complete retinal measures at visit 5 (N=1345) are provided in Table S8. Significantly more individuals had greater WMH burden (CHS >2) at visit 5 (60%) than at visit 3 (9.9%). The differences in late‐life clinical characteristics between individuals with greater WMH burden and those with little or no WMH burden were similar to visit 3. Participants with greater WMH burden (compared with those with little or no WMH) had a higher prevalence of retinopathy (6.6% versus 3.4%), arteriovenous nicking (7.5% versus 3.9%), and hypertension (76% versus 66%) and were more likely to belong to the older age category, age 78 years or older (43% versus 21%). Individuals with significant WMH burden at visit 5 were also more likely to belong to the severe prescreening categories for all 3 prescreens.

### Recruitment Estimation in Late Life With Unweighted and Weighted Scores

Most individuals in late life had significant WMH burden (CHS >2) (60%), a rate that was significantly higher than the prevalence of WMH burden in late midlife (9.9%) (Table S8). Therefore, to achieve the trial's target sample size (*N*=646), a significantly lower number of participants (N=1077) in late life than in late midlife (*N*=6526) would need to be enrolled in an MRI‐only recruitment study (Table S9). Thus, the associated theoretical recruitment costs would be 6 times lower at late life ($700 050) than in late midlife ($4 241 900).

Employing any of the unweighted retinal or clinical prescreening modalities did not significantly reduce the overall recruitment costs in late life as compared with the MRI‐only recruitment approach (Table S9). We drew similar conclusions when using the weighted instead of the unweighted measures (Table S10; Figure S6). The weights assigned to the retinal and clinical individual prescreening features in late life can be found in Table S11.

## DISCUSSION

This study demonstrates that a 2‐stage recruitment design with a low‐cost prescreening measure is a promising approach for a phase‐2 clinical trial in SVD, resulting in reduced theoretical recruitment costs compared with an MRI‐only design in individuals who are at increased risk for WMH presence and its progression in late midlife. Whether considering a retinal prescreen, a clinical prescreen, or a combination, recruitment costs were consistently lower when selecting participants with more severe score burden within each of those schemas. Longitudinally, we found consistent predictive value of the high retinal and clinical scores for WMH progression. Our study additionally emphasizes that the 2‐stage design has particular value in late midlife but not in late life when the prevalence of significant WMH burden, at least in the range we explored here, is significantly higher. A study in late life might, however, be more likely to target a more stringent/more severe WMH cutoff. In this scenario, it is quite possible that a 2‐stage design would still be useful in reducing costs. Finally, recognizing the potential value of the 2‐stage approach beyond the field of cerebrovascular disease burden, we created a user‐friendly publicly available program (see link in Methods). This 2‐stage design could be used in the field of cardiovascular diseases, cancer, and metabolic diseases in contexts where only a fraction of the population would be eligible for clinical trial enrollment and where a less expensive marker is available that could be used as a prescreening measure.^28^, ^29^, ^30^, ^31^

The reduction of costs associated with the 2‐stage design has several implications. First, the reduced cost would make prevention trials more feasible. Second, the design would create a window of opportunity to test the effectiveness of interventions even in a preclinical stage of disease. A limitation of existing clinical trials has been that they often target an adult population in late life (age 70s or 80s), which may represent a disease stage too advanced for potential intervention or prevention. It is quite possible that disease‐modifying treatments are more useful in earlier disease stages and that unsuccessful therapeutic interventions previously tested in older adults may be more effective in individuals with significant WMH burden in late midlife.^12^

Several lines of evidence have shown that risk factors in midlife are significantly associated with cerebrovascular disease and late‐life clinical outcomes. In the ARIC cohort, cumulative blood pressure over a 10‐year period in midlife was a significant predictor for WMH progression.^18^ Given the importance of risk factors in midlife, we hope that introducing a 2‐stage design will resolve the currently seen bottleneck characterized by multiple proposed risk factor interventions but with very few clinical trials on WMH progression in late midlife.

To our knowledge, this is the first comprehensive study in the field of cerebrovascular disease investigating how to reduce the recruitment cost for a clinical trial in a midlife population. The findings of this theoretical recruitment study are promising showing that a prescreening assessment significantly reduces cost for MRI screening by more than 1 million. We anticipate that innovative machine‐learning approaches on low‐cost measures could further reduce recruitment costs for clinical trials in cerebrovascular disease in the future.^32^, ^33^ We think that these machine‐learning approaches could particularly be effective in reducing the high FP rates we observed when selecting a wide prescreening (mild–severe) range and when aiming for a high TP rate. On the other hand, machine‐learning approaches may also be powerful when choosing a severe prescreening score range, which has been shown to be associated with the lowest recruitment cost in this study, by significantly increasing the TP rate while keeping the FP rate at a low level. We first saw evidence of this potential when comparing the TP rate of the weighted to the unweighted scores. Choosing a severe weighted score significantly increased the TP rate across all 3 prescreening modalities when compared with the same unweighted score range (Table S2; Figure S4). The impact on the recruitment size was profound showing that more than 10 000 individuals were not needed when selecting the severe weighted instead of the severe unweighted retinal or clinical score range (Table 2; Table S7). Ultimately, a very high TP rate paired with a very low FP rate should be the goal to substantively reduce the recruitment sample size and recruitment costs in a 2‐stage design.

The study also has some limitations. First, we estimated the standard retinal and MRI costs to be $32.50 and $650 respectively. However, cost estimates can significantly vary and may limit the results to the United States. We also assumed $0 cost of identifying an individual who is willing to be screened based on already available clinical information. Second, we did not assign a cost for identifying, contacting, and scheduling participants for the assessments. Third, we acknowledge that age 60 years as a cutoff for the clinical prescreening score may appear a bit arbitrary. We chose the cutoff based on previous evidence showing that around 10% to 20% of asymptomatic individuals around 60 years of age have WMH burden.^34^, ^35^ Because visit 5 (2011–2013) was approximately 18 years after visit 3 (1993–1995), age 78 years was selected as the cutoff in late life. Fourth, we also recognize that although the ARIC cohort is meant to be representative of 4 US communities, it may not reflect expected rates in a clinical trial population recruited from other communities. Fifth, a significant proportion of individuals who had missing or ungradable retinal measures had to be excluded from the study. Excluded participants were significantly older, more likely to be of Black race, and to have diabetes and hypertension, which may have lowered the proportions of individuals who had a positive prescreening feature and significant WMH lesion burden. Sixth, both WMH graded and WMH volume measures were used for the analysis, and despite our best efforts to replicate the analysis in midlife and late life as closely as possible, we cannot entirely rule out the possibility that differences in WMH assessment could have influenced the recruitment sample size estimates. Seventh, not all individuals who had undergone MRI assessment in late midlife were also enrolled in the imaging study in late life. It is possible that differences in the sample composition may have also affected the recruitment estimates. Participants with retinal microvascular burden and significant WMH burden in late life may have been less likely to undergo MRI scanning, which could have led to an underestimation of the overall disease burden in the population. In this recruitment study, the underestimation could have resulted in higher recruitment sample size estimates for the hypothetical clinical trial than needed. On the other hand, more conservative recruitment estimates may actually be required as they are more reflective of the healthier population enrolled in clinical trials.^36^, ^37^ Future studies should address these limitations and further validate these prescreening measures. Eighth, we provide a variety of performance measures, which allows the reader to compare the different prescreening modalities and severity thresholds (Table S2). It is, however, important to point out that the positive predictive value and negative predictive value can be affected by the underlying prevalence estimates.

### Conclusions

In conclusion, a 2‐stage recruitment design may be a promising approach to reduce the overall costs of clinical trials in late midlife. As the population is rapidly aging and as the vascular burden is increasing globally, there is a real urgency to test new therapies aiming to slow down WMH progression starting in late midlife. Consideration of adding a prescreen stage to clinical recruitment may allow for more efficient enrollment into clinical trials in this area and in other areas dependent on an expensive screening measure such as MRI.

## Sources of Funding

The ARIC Study is carried out as a collaborative study supported by National Heart, Lung, and Blood Institute (NHLBI) contracts 75N92022D00001, 75N92022D00002, 75N92022D00003, 75N92022D00004, 75N92022D00005. The ARIC Neurocognitive Study is supported by U01HL096812, U01HL096814, U01HL096899, U01HL096902, and U01HL096917 from the NIH (NHLBI, National Institute of Neurological Disorders and Stroke, National Institute on Aging, and National Institute of Deafness and Other Communication Disorders) with MRI examinations funded by NIH R01‐HL70825. Marco Egle, Mohini Johri, and Rebecca F. Gottesman were supported by the National Institute of Neurological Disorders and Stroke Intramural Research Program.

## Disclosures

None.

## Supporting information

Data S1–S2
Tables S1–S11
Figures S1–S6
