# Supplementary material for: Two‐Stage Recruitment Design to Reduce Magnetic Resonance Imaging Screening Cost for a Theoretical Clinical Trial of White Matter Hyperintensity Progression
Source: J Am Heart Assoc. 2024 Nov 15;13(22):e036140. doi: 10.1161/JAHA.124.036140 (PMC11681408; doi:10.1161/JAHA.124.036140)
Supplement: Supplementary file 1 — Data S1–S2 Tables S1–S11 Figures S1–S6 [file JAH3-13-e036140-s001.pdf]

Supplemental Material

Supplemental Methods

Data S1. Computation of the weighted pre-screening scores

As for the unweighted scoring system, the weighted scoring system ranged from 0-3 indicating the degree of retinal and/or clinical burden per participant. To generate the weights per pre-screening feature, a ridge logistic regression model was used with the clinical or retinal measures as predictors and the WMH burden as dichotomous outcome (CHS>2 vs.≤2). The weights for each pre-screening feature were derived from the regression coefficients and normalized to a score range of 0-3. To generalize the weights beyond the sample of our study, the optimal lambda value of the regularized method was chosen that minimized the cross-validation prediction error rate for WMH burden. The weighted clinical-retinal scores were created using the sum of the normalized weighted retinal and clinical scores and truncated to a maximum score of 3. The clinical and clinical-retinal weighted scores were rounded and were labeled the same way as for the unweighted scores. Due to distributional differences, the weighted retinal scores were graded differently as follows: none (score= 0), mild (0 <score< 0.5); moderate (0.5< score< 1); severe (1 <score≤ 3). Pearson's Chi-squared tests were used to test for differences in retinal and clinical scores by significant WMH burden.

Data S2: User interface of the 2-stage recruitment calculator

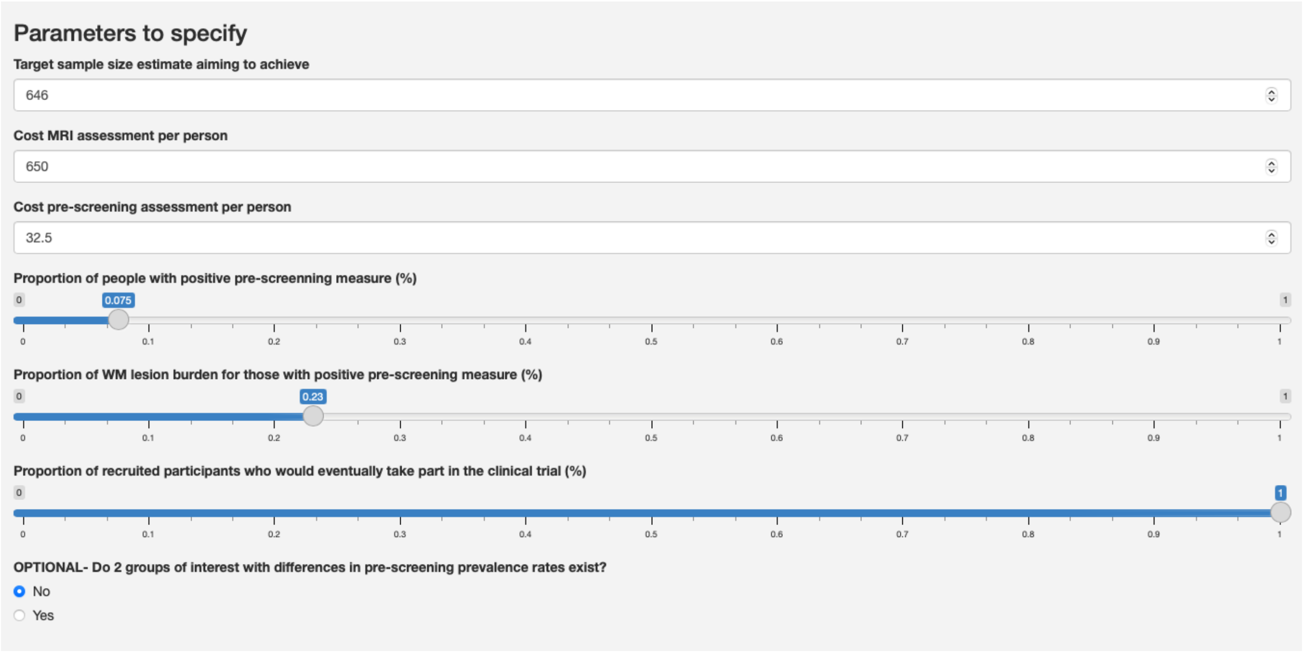

WM- White Matter

Table S1. Comparing clinical characteristics at baseline between those individuals included vs. excluded in the study

**Characteristic**	**Overall**, N = 1,925	**included**, N = 1,311	**excluded**, N = 614	**p-value**‡
Age *	62.0 (59.0, 66.0)	61.0 (58.0, 65.0)	64.0 (60.0, 67.8)	<0.001
Sex†				>0.9
Female	1,152 (60%)	785 (60%)	367 (60%)	
Race†				<0.001
Black	960 (50%)	596 (45%)	364 (59%)	
Diabetes†				0.012
present	349 (18%)	219 (17%)	130 (22%)	
Unknown	14	4	10	
Hypertension†				0.015
present	936 (49%)	613 (47%)	323 (53%)	
Unknown	13	8	5	

*Median(Q1, Q3),
†n (%),
‡ Pearson's Chi-squared tests

Table S2. The various performance metrics given the different severity thresholds from the three unweighted and weighted pre-screening modalities

Scores	Pre-screening modality	Severity range	True Positive (TP) Rate	False Positive (FP) Rate	Specificity	Positive predictive value (PPV)	Negative predictive value (NPV)
Unweighted	Retinal	Severe	0.18	0.06	0.94	0.24	0.91
Unweighted	Retinal	Moderate-severe	0.25	0.11	0.89	0.19	0.91
Unweighted	Retinal	Mild-severe	0.54	0.39	0.61	0.13	0.92
Unweighted	Clinical	Severe	0.22	0.17	0.83	0.13	0.91
Unweighted	Clinical	Moderate-severe	0.61	0.37	0.63	0.15	0.94
Unweighted	Clinical	Mild-severe	0.95	0.80	0.20	0.12	0.98
Unweighted	Clinical-retinal	Severe	0.48	0.26	0.74	0.17	0.93
Unweighted	Clinical-retinal	Moderate-severe	0.76	0.55	0.45	0.13	0.95
Unweighted	Clinical-retinal	Mild-severe	0.99	0.86	0.14	0.11	0.99
Weighted	Retinal	Severe	0.28	0.11	0.90	0.23	0.92
Weighted	Retinal	Moderate-severe	0.41	0.20	0.80	0.18	0.93
Weighted	Retinal	Mild-severe	0.54	0.39	0.61	0.13	0.92
Weighted	Clinical	Severe	0.54	0.30	0.70	0.17	0.93
Weighted	Clinical	Moderate-severe	0.61	0.37	0.63	0.15	0.94
Weighted	Clinical	Mild-severe	0.94	0.78	0.22	0.11	0.97
Weighted	Clinical-retinal	Severe	0.66	0.35	0.65	0.17	0.95
Weighted	Clinical-retinal	Moderate-severe	0.72	0.45	0.55	0.15	0.95
Weighted	Clinical-retinal	Mild-severe	0.96	0.80	0.20	0.12	0.98

Table S3. Change in unweighted pre-screening measure's total recruitment sample size and cost by attrition rate

Pre-screening modality	Unweighted summary scores	Attrition rate	Total recruitment sample size	Total Cost
Retinal	Severe	None	37,450	$ 3,042,975
Retinal	Moderate-severe	None	26,154	$ 3,060,655
Retinal	Mild-severe	None	12,424	$ 3,634,280
Retinal	Severe	10%	41,611	$ 3,381,008
Retinal	Moderate-severe	10%	29,060	$ 3,400,150
Retinal	Mild-severe	10%	13,804	$ 4,037,930
Retinal	Severe	20%	46,812	$ 3,803,540
Retinal	Moderate-severe	20%	32,693	$ 3,825,672
Retinal	Mild-severe	20%	15,529	$ 4,542,492
Retinal	Severe	30%	53,499	$ 4,347,168
Retinal	Moderate-severe	30%	37,363	$ 4,371,998
Retinal	Mild-severe	30%	17,748	$ 5,191,810
Clinical	Severe	None	52,778	$ 2,470,650
Clinical	Moderate-severe	None	10,767	$ 2,799,550
Clinical	Mild-severe	None	6,566	$ 3,500,250
Clinical	Severe	10%	58,642	$ 2,744,950
Clinical	Moderate-severe	10%	11,963	$ 3,110,900
Clinical	Mild-severe	10%	7,295	$ 3,888,300
Clinical	Severe	20%	65,973	$ 3,088,150
Clinical	Moderate-severe	20%	13,459	$ 3,499,600
Clinical	Mild-severe	20%	8,207	$ 4,374,500
Clinical	Severe	30%	75,397	$ 3,528,850
Clinical	Moderate-severe	30%	15,381	$ 3,999,450
Clinical	Mild-severe	30%	9,379	$ 4,999,150
Clinical-retinal	Severe	None	13,572	$ 2,911,740
Clinical-retinal	Moderate-severe	None	8,718	$ 3,513,835
Clinical-retinal	Mild-severe	None	6,751	$ 4,037,508
Clinical-retinal	Severe	10%	15,080	$ 3,235,050
Clinical-retinal	Moderate-severe	10%	9,687	$ 3,904,128
Clinical-retinal	Mild-severe	10%	7,501	$ 4,485,682
Clinical-retinal	Severe	20%	16,965	$ 3,639,512
Clinical-retinal	Moderate-severe	20%	10,898	$ 4,391,985
Clinical-retinal	Mild-severe	20%	8,438	$ 5,046,535
Clinical-retinal	Severe	30%	19,388	$ 4,158,960
Clinical-retinal	Moderate-severe	30%	12,455	$ 5,019,788
Clinical-retinal	Mild-severe	30%	9,644	$ 5,767,580

Table S4. Median change in WMH volume over 10-years by retinal, clinical, and clinical-retinal score severity

**Unweighted score**	**N (%)**	**Median WMH progression (cm^3), (Q1, Q3)**
Retinal score		
None	456 (62%)	2.22 (-0.34, 5.97)
Mild	197 (27%)	2.25 (-0.32, 7.94)
Moderate	39 (5.3%)	3.42 (1.17, 7.91)
Severe	39 (5.3%)	5.30 (1.83, 10.05)
Clinical score		
None	151 (21%)	1.11 (-1.21, 3.22)
Mild	332 (45%)	2.14 (-0.42, 6.45)
Moderate	216 (30%)	3.55 (1.17, 8.73)
Severe	32 (4.4%)	6.23 (0.80, 15.54)
Clinical-retinal score		
None	107 (15%)	0.77 (-1.21, 3.08)
Mild	250 (34%)	1.98 (-0.67, 5.10)
Moderate	210 (29%)	2.67 (0.44, 8.06)
Severe	164 (22%)	4.64 (1.13, 9.84)

WMH- White Matter Hyperintensities

Table S5. Weights given to each individual pre-screening feature in late midlife

Features	Normalized weights (w)
Retinal measures	
Retinopathy	1.210
Arteriovenous nicking	1.187
Focal arteriolar narrowing	0.583
Generalized arteriolar narrowing	0.019
Clinical measures	
Age above 60 years	1.354
Hypertension	1.314
Diabetes	0.331

Table S6. Features of the weighted summary scores in late midlife, by WMH category

Characteristics	Overall, N= 1311*	No or low WMH burden, N= 1181*	Significant WMH burden, N= 130*	p-value†
**Weighted summary scores‡**				
Retinal score				**<0.001**
None	781 (60%)	721 (61%)	60 (46%)	
Mild	241 (18%)	224 (19%)	17 (13%)	
Moderate	128 (9.8%)	112 (9.5%)	16 (12%)	
Severe	161 (12%)	124 (10%)	37 (28%)	
Clinical score				**<0.001**
None	267 (20%)	259 (22%)	8 (6.2%)	
Mild	524 (40%)	481 (41%)	43 (33%)	
Moderate	95 (7.2%)	86 (7.3%)	9 (6.9%)	
Severe	425 (32%)	355 (30%)	70 (54%)	
Clinical-retinal score				**<0.001**
None	238 (18%)	233 (20%)	5 (3.8%)	
Mild	444 (34%)	413 (35%)	31 (24%)	
Moderate	128 (10%)	120 (10%)	8 (6.9%)	
Severe	501 (38%)	415 (35%)	86 (65%)	

* n (%);

† Pearson's Chi-squared tests;

‡ Score categories: none=0; mild=1, moderate= 2, severe= 3 or highest score

Table S7. Estimated sample size for a theoretical recruitment study by each of the weighted screening approaches in late midlife

Theoretical inclusion criteria	Overall		1. Pre-screening stage *Number of participants at pre-screening stage and associated cost (proportion of those individuals with a positive pre-screening feature)*		2. MRI screening stage *Number of participants at the MRI screening stage and associated cost (proportion of those individuals with significant WMH burden)*	
**Retinal score range**	Sample size	Financial cost	Sample size	Financial cost	Sample size	Financial cost
Severe	23,406	$ 2,586,545	23,406 (12%)	$ 760,695	2,809 (23%)	$ 1,825,850
Moderate-severe	16,314	$ 2,863,705	16,314 (22%)	$ 530,205	3,590 (18%)	$ 2,333,500
Mild- severe	12,424	$ 3,634,280	12,424 (40%)	$ 403,780	4,970 (13%)	$ 3,230,500
**Clinical score range**						
Severe	12,618	$ 2,624,700	12,618 (32%)	$ 0	4,038 (16%)	$ 2,624,700
Moderate-severe	11,043	$ 2,799,550	11,043 (39%)	$ 0	4,307 (15%)	$ 2,799,550
Mild- severe	6,730	$ 3,499,600	6,730 (80%)	$ 0	5,384 (12%)	$ 3,499,600
**Clinical-retinal score range**						
Severe	10,000	$ 2,795,000	10,000 (38%)	$ 325,000	3,800 (17%)	$ 2,470,000
Moderate-severe	8,973	$ 3,091,822	8,973 (48%)	$ 291,623	4,308 (15%)	$ 2,800,200
Mild- severe	6,566	$ 3,713,645	6,566 (82%)	$ 213,395	5,385 (12%)	$ 3,500,250
**No prescreening**						
MRI only	6,526	$ 4,241,900	-	-	6,526 (9.9%)	$ 4,241,900

MRI- Magnetic Resonance Imaging; WMH – White Matter Hyperintensities

Table S8. Features of the individual measures and summary scores in late life, by WMH category

Characteristics	Overall, N= 1,345	No or low WMH burden, N= 545	Significant WMH burden, N= 800	p-value[‡]
Age*	76 (72, 80)	74 (71, 78)	77 (74, 81)	**< 0.001**
**Retinal measures[†]**				
Retinopathy	73 (5.4%)	20 (3.7%)	53 (6.6%)	**0.019**
Arteriovenous nicking	81 (6.0%)	21 (3.9%)	60 (7.5%)	**0.006**
Focal arteriolar narrowing	58 (4.3%)	17 (3.1%)	41 (5.1%)	0.075
Generalized arteriolar narrowing	369 (27%)	135 (25%)	234 (29%)	0.071
Demographic and clinical measures				
Age 78 or older	458 (34%)	113 (21%)	345 (43%)	**< 0.001**
Diabetes	415 (31%)	162 (30%)	253 (32%)	0.5
Hypertension	970 (72%)	361 (66%)	609 (76%)	**< 0.001**
**Unweighted summary scores[§]**				
Retinal score				**0.001**
None	835 (62%)	369 (68%)	466 (58%)	
Mild	392 (29%)	144 (26%)	248 (31%)	
Moderate	43 (3.2%)	12 (2.2%)	31 (3.9%)	
Severe	75 (5.6%)	20 (3.7%)	55 (6.9%)	
Clinical score				**< 0.001**
None	206 (15%)	122 (22%)	84 (10%)	
Mild	554 (41%)	236 (43%)	318 (40%)	
Moderate	466 (35%)	161 (30%)	305 (38%)	
Severe	119 (8.8%)	26 (4.8%)	93 (12%)	
Clinical-retinal score				**< 0.001**
None	139 (10%)	86 (16%)	53 (6.6%)	
Mild	414 (31%)	194 (36%)	220 (28%)	
Moderate	458 (34%)	174 (32%)	284 (36%)	
Severe	334 (25%)	91 (17%)	243 (30%)	
**Weighted summary scores[§]**				
Retinal score				**< 0.001**
None	835 (62%)	369 (68%)	466 (58%)	

Mild	314 (23%)	120 (22%)	194 (24%)	
Moderate	131 (9.7%)	39 (7.2%)	92 (12%)	
Severe	65 (4.8%)	17 (3.1%)	48 (6.0%)	
Clinical score				**< 0.001**
None	259 (19%)	151 (28%)	108 (14%)	
Mild	628 (47%)	281 (52%)	347 (43%)	
Moderate	116 (8.6%)	33 (6.1%)	83 (10%)	
Severe	342 (25%)	80 (15%)	262 (33%)	
Clinical-retinal score				**< 0.001**
None	234 (17%)	139 (26%)	95 (12%)	
Mild	576 (43%)	258 (47%)	318 (40%)	
Moderate	174 (13%)	67 (12%)	107 (13%)	
Severe	361 (27%)	81 (15%)	280 (35%)	

*Median(Q1, Q3); †n(%); ‡Wilcoxon rank sum test, Pearson's Chi-squared test comparing significant vs no or low WMH burden; §Score categories: none=0; mild=1, moderate= 2, severe= 3 or highest score

WMH- White Matter Hyperintensities

Table S9. Estimated sample size for a theoretical recruitment study by each of the unweighted screening approaches in late life

Theoretical inclusion criteria	Overall		1. Pre-screening stage *Number of participants at pre-screening stage and associated cost (proportion of those individuals with a positive pre-screening feature)*		2. MRI screening stage *Number of participants at the MRI screening stage and associated cost (proportion of those individuals with significant WMH burden)*	
**Retinal score range**	Sample size	Financial cost	Sample size	Financial cost	Sample size	Financial cost
Severe only	15,803	$ 1,088,848	15,803 (5.6%)	$ 513,598	885 (73%)	$ 575,250
Moderate-severe	10,172	$ 905,840	10,172 (8.7%)	$ 330,590	885 (73%)	$ 575,250
Mild- severe	2,616	$ 731,770	2,616 (38%)	$ 85,020	995 (65%)	$ 646,750
**Clinical score range**						
Severe only	9,412	$ 538,850	9,412 (8.8%)	$ 0	829 (78%)	$ 538,850
Moderate-severe	2,160	$ 618,150	2,160 (44%)	$ 0	951 (68%)	$ 618,150
Mild- severe	1,207	$ 666,900	1,207 (85%)	$ 0	1,026 (63%)	$ 666,900
**Clinical-retinal score range**						
Severe only	3,540	$ 690,300	3,540 (25%)	$ 115,050	885 (73%)	$ 575,250
Moderate-severe	1,635	$ 680,388	1,635 (59%)	$ 53,138	965 (67%)	$ 627,250
Mild- severe	1,158	$ 715,585	1,158 (90%)	$ 37,635	1,043 (62%)	$ 677,950
**No prescreening**						
MRI only	1,077	$ 700,050	-	-	1,077 (60%)	$ 700,050

MRI- Magnetic Resonance Imaging; WMH- White Matter Hyperintensities

Table S10. Estimated sample size for a theoretical recruitment study by each of the weighted screening approaches in late life

**Theoretical inclusion criteria**	**Overall**		**1. Pre-screening stage** *Number of participants at pre-screening stage and associated cost (proportion of those individuals with a positive pre-screening feature)*		**2. MRI screening stage** *Number of participants at the MRI screening stage and associated cost (proportion of those individuals with significant WMH burden)*	
**Retinal score range**	Sample size	Financial cost	Sample size	Financial cost	Sample size	Financial cost
Severe only	18,187	$ 1,158,528	18,187 (4.8%)	$ 591,078	873 (74%)	$ 567,450
Moderate-severe	6,066	$ 788,645	6,066 (15%)	$ 197,145	910 (71%)	$ 591,500
Mild- severe	2,616	$ 731,770	2,616 (38%)	$ 85,020	995 (65%)	$ 646,750
**Clinical score range**						
Severe only	3,356	$ 545,350	3,356 (25%)	$ 0	839 (77%)	$ 545,350
Moderate-severe	2,534	$ 560,300	2,534 (34%)	$ 0	862 (75%)	$ 560,300
Mild- severe	1,247	$ 657,150	1,247 (81%)	$ 0	1011 (64%)	$ 657,150
**Clinical-retinal score range**						
Severe only	3,068	$ 638,560	3,068 (27%)	$ 99,710	829 (78%)	$ 538,850
Moderate-severe	2,244	$ 656,630	2,244 (40%)	$ 72,930	898 (72%)	$ 583,700
Mild- severe	1,236	$ 707,070	1,236 (83%)	$ 40,170	1,026 (63%)	$ 666,900
**No prescreening**						
MRI only	1,077	$ 700,050	-	-	1,077 (60%)	$ 700,050

MRI- Magnetic Resonance Imaging; WMH- White Matter Hyperintensities

Table S11. Weights given to each individual pre-screening feature in late life

Features	Normalized weights (w)
Retinal measures	
Retinopathy	0.91
Arteriovenous nicking	1.00
Focal arteriolar narrowing	0.74
Generalized arteriolar narrowing	0.35
Clinical measures	
Age above 78 years	2.03
Hypertension	0.89
Diabetes	0.09

Figure S1. Overview about the ARIC study design and the WMH measures available

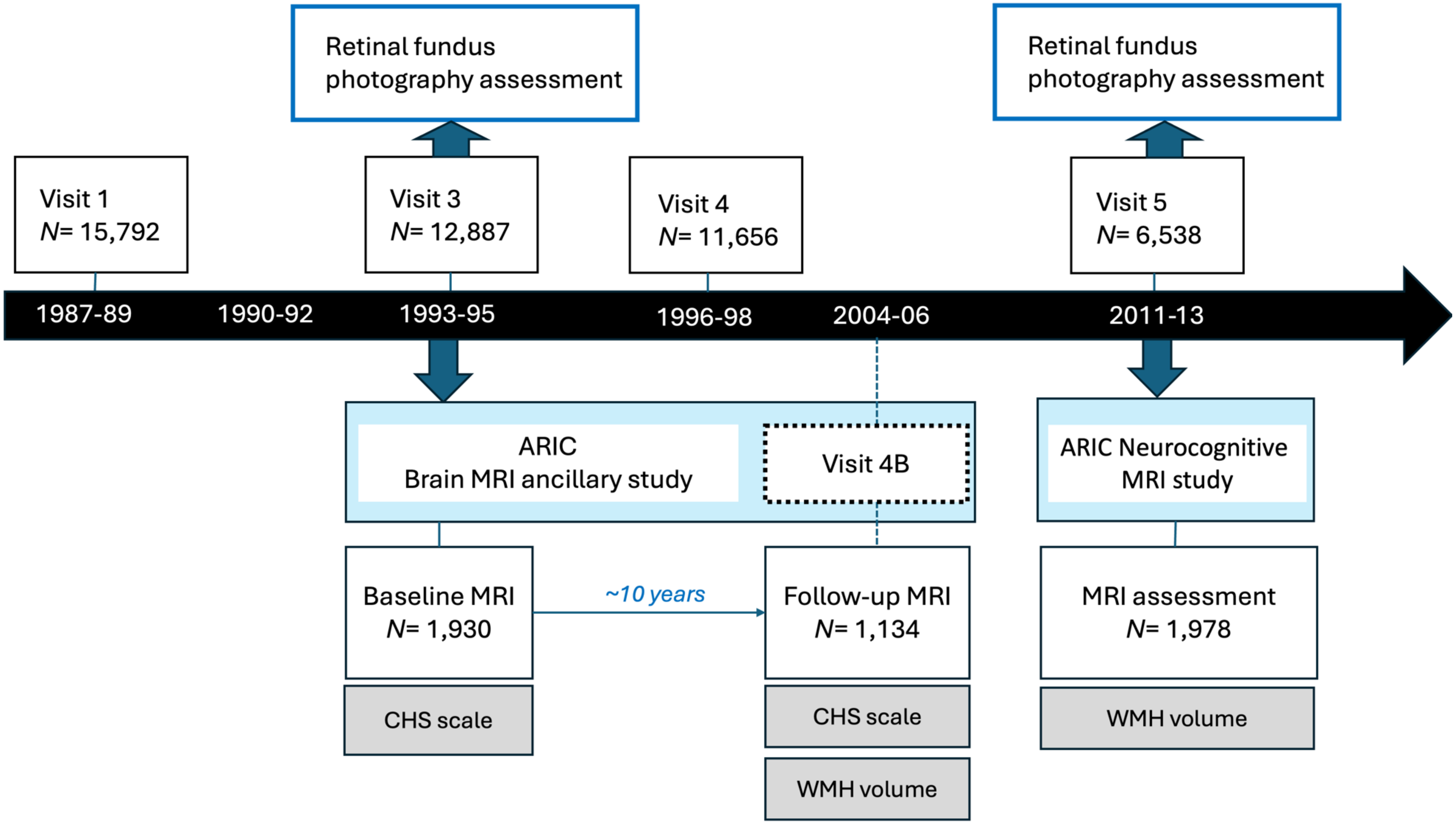

ARIC- Atherosclerosis Risk in Communities; CHS- Cardiovascular Health Study; MRI- Magnetic Resonance Imaging; WMH- White Matter Hyperintensities

Figure S2. Graphical illustration demonstrating how the weighted scores were derived

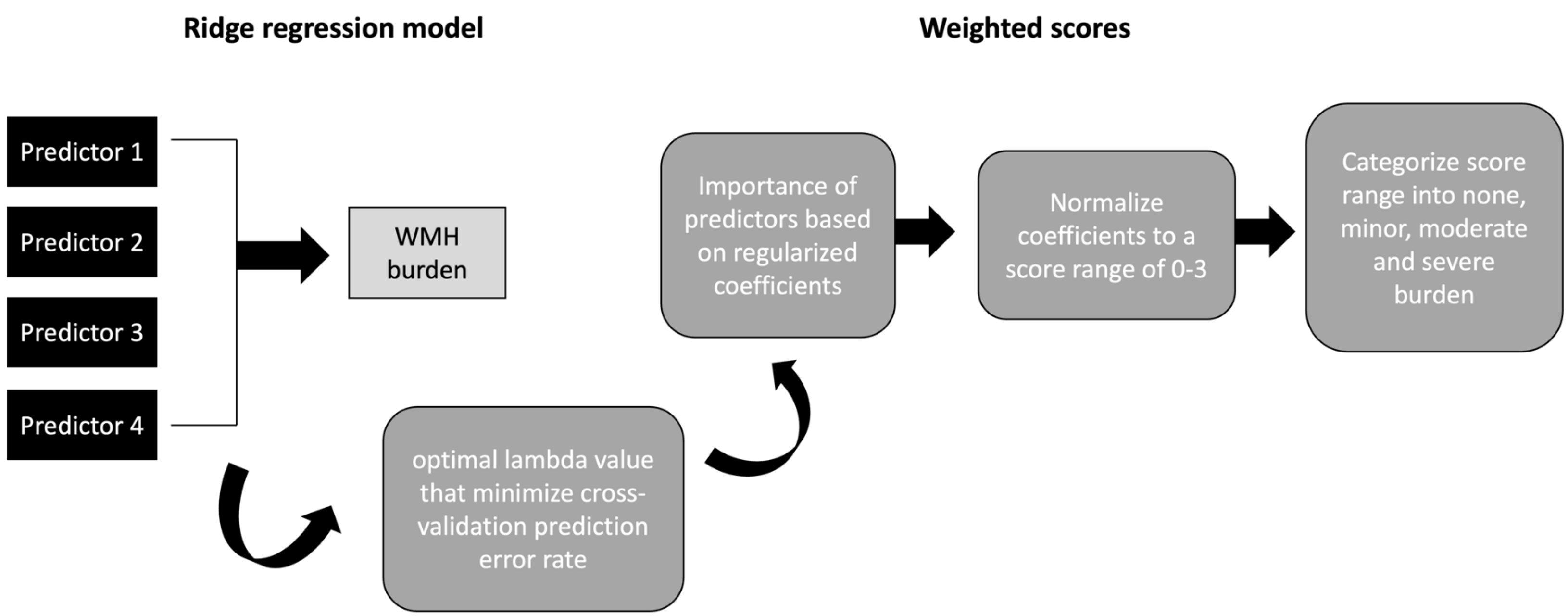

WMH- White Matter Hyperintensities

Figure S3. Flowchart of the sample used to complete the analysis in midlife

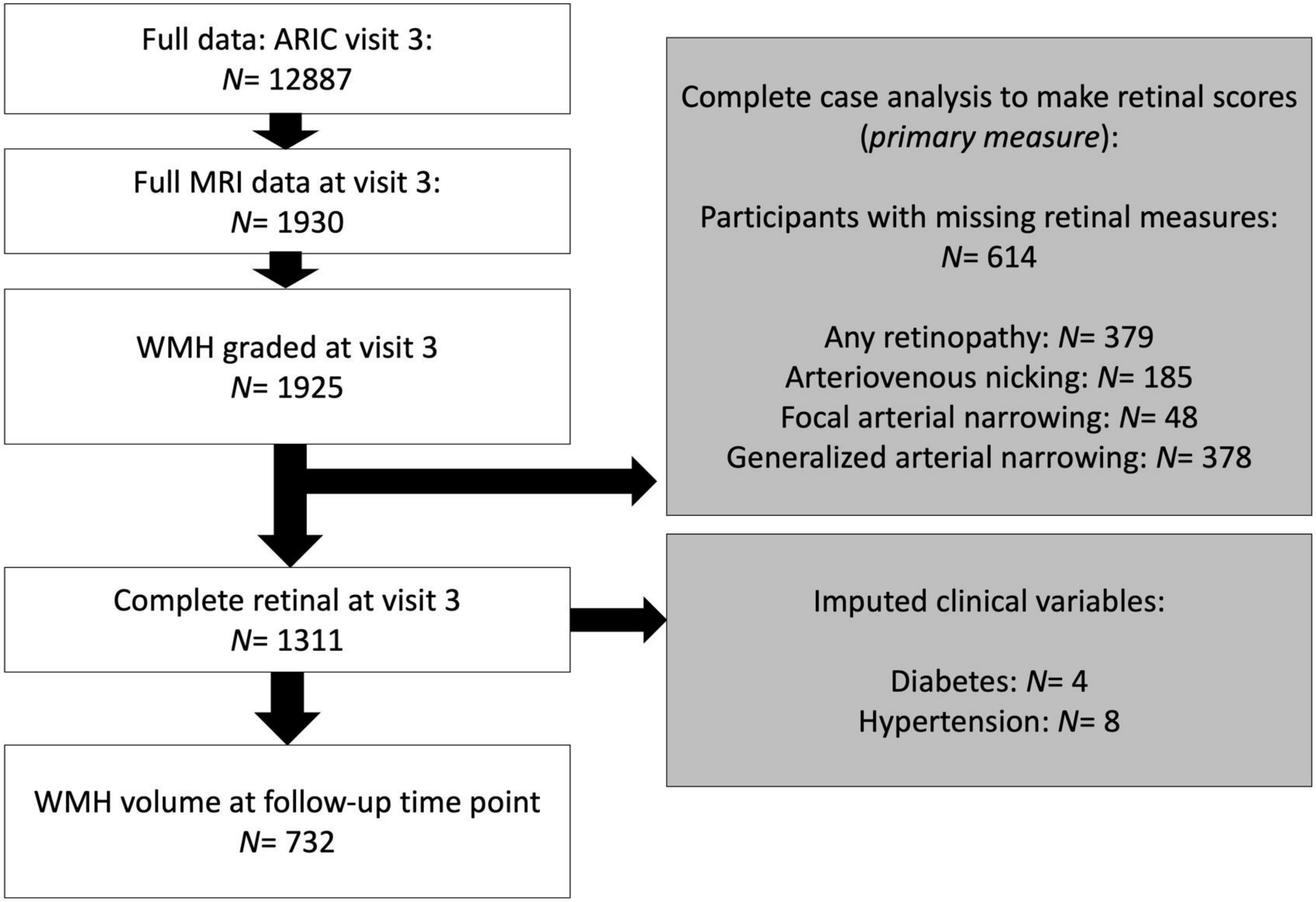

ARIC- Atherosclerosis Risk in Communities; MRI- Magnetic Resonance Imaging; WMH- White Matter Hyperintensities

Figure S4. Bar chart showing the different pre-screening score ranges on significant vs. no or little WMH burden

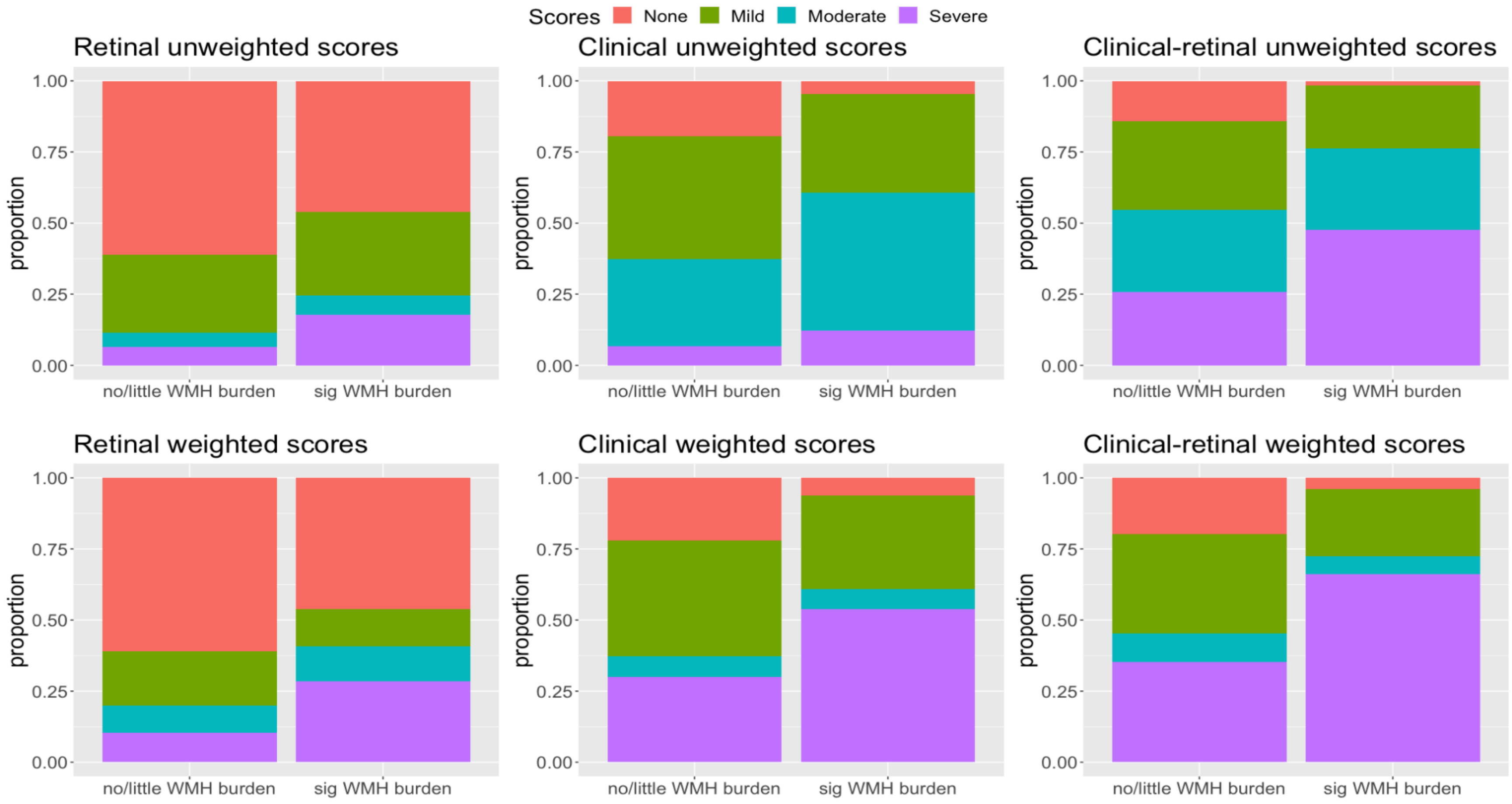

WMH- White Matter Hyperintensities

Figure S5. Sample size estimates and costs for all unweighted and weighted late midlife scores

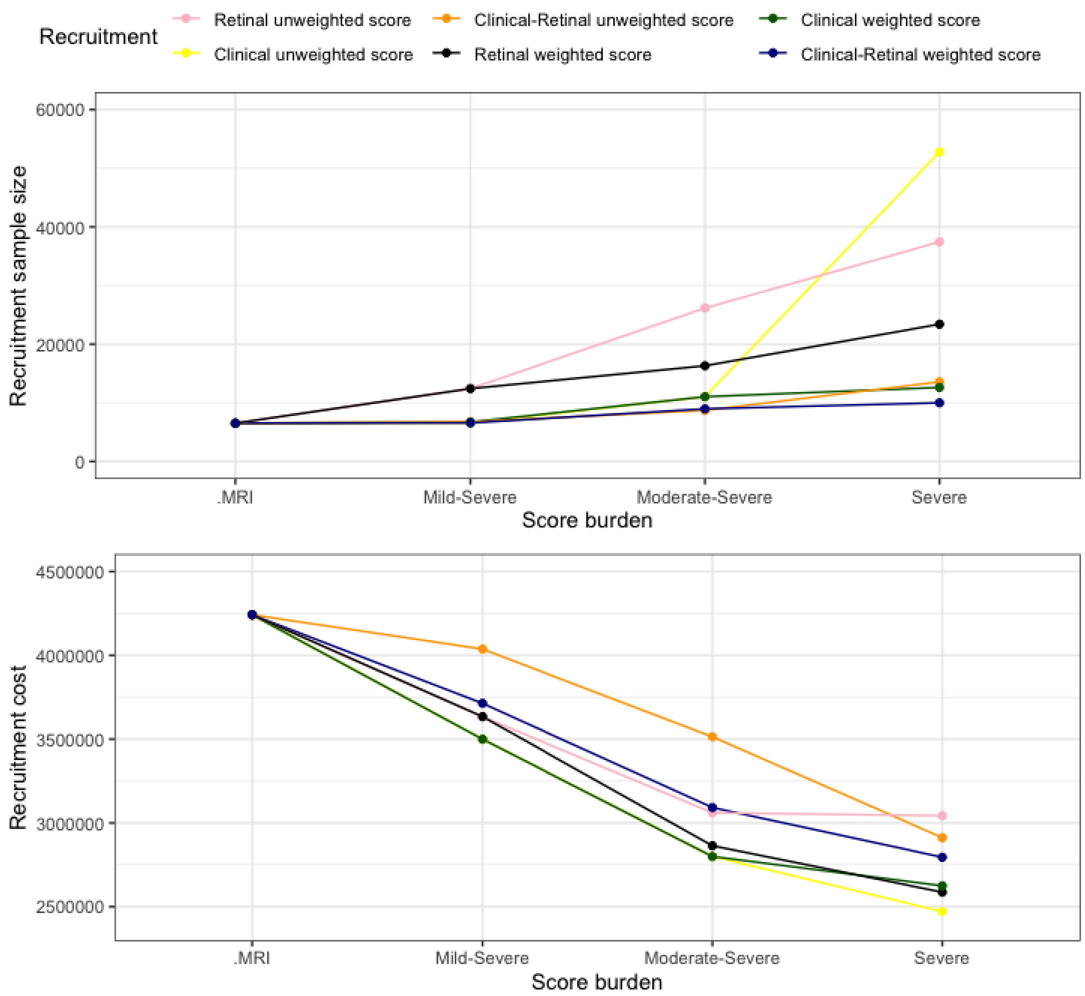

MRI- Magnetic Resonance Imaging

Figure S6. Total recruitment sample size and cost for various score cutoffs for each of the recruitment modalities in late life

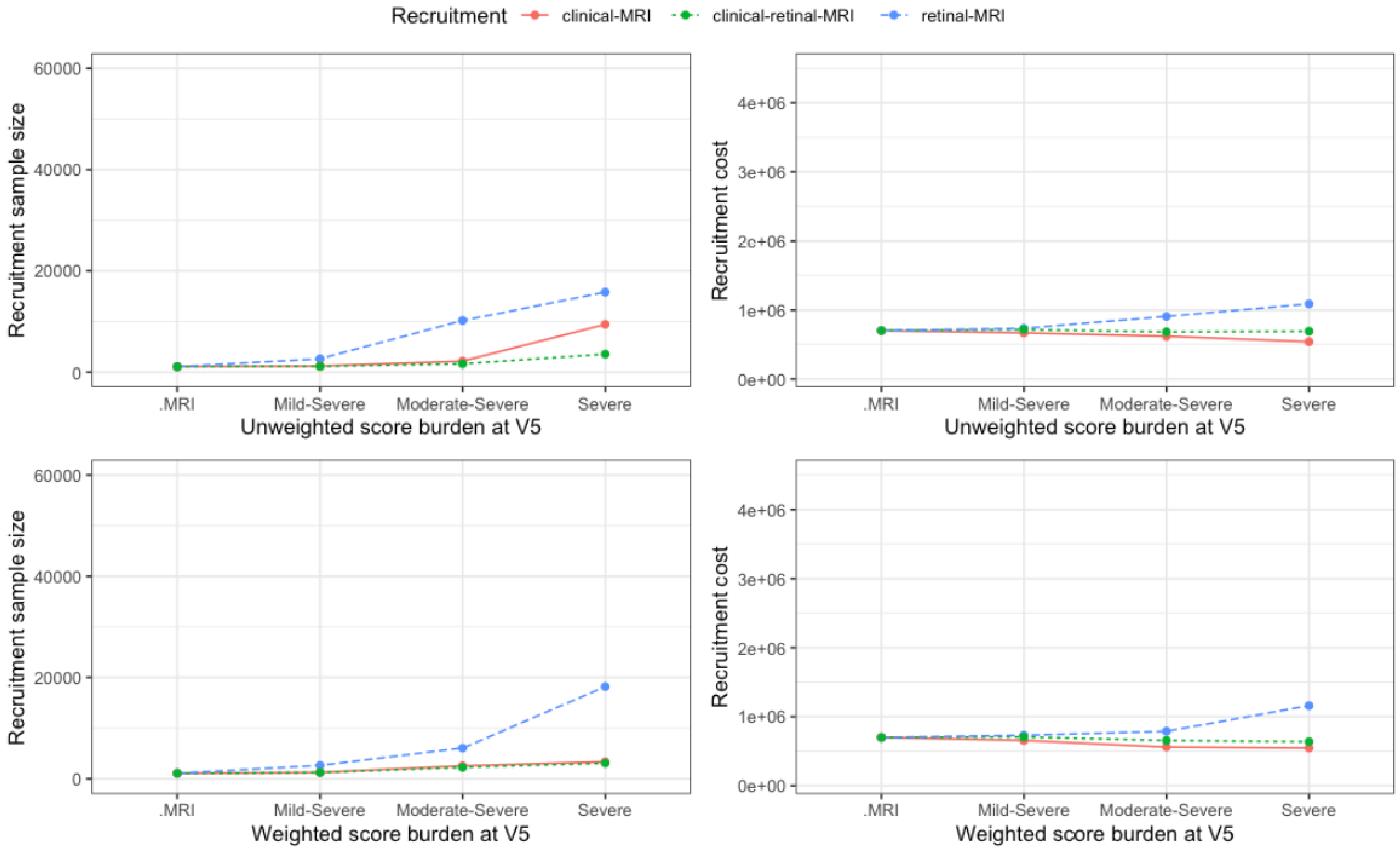

MRI- Magnetic Resonance Imaging
